# The Interaction between Iron Cyanide and Lead Ions in Pyrite Flotation

**DOI:** 10.3390/molecules29112517

**Published:** 2024-05-27

**Authors:** Xiaoxia Yang, Yufan Mu, Shiqi Liu

**Affiliations:** 1 School of Mechanical Engineering, Taiyuan University of Science and Technology, Taiyuan 030024, China; 2 The University of Queensland, Brisbane, QLD 4072, Australia; yufan.mu@molycop.com; 3 Molycop Innovation, Molycop, Omaha, NE 68106, USA; 4 College of Mining Engineering, Taiyuan University of Technology, Taiyuan 030024, China; liushiqi01@tyut.edu.cn

**Keywords:** pyrite flotation, iron cyanide depression, lead activation, surface species, EIS

## Abstract

Despite being a major cyanide species in the process water, it is unclear how iron cyanide influences pyritic gold ore flotation as well as how lead ions influence pyritic gold ore flotation in the presence of iron cyanide. This study aims at revealing the interaction of Fe(CN)_6_^3−^ and lead ions in pyrite flotation to investigate the strong depressing effect of Fe(CN)_6_^3−^ on pyritic gold ore flotation and the significant activating effect of lead ions on pyritic gold ore flotation in the presence of Fe(CN)_6_^3−^ using flotation, zeta potential measurement and surface analysis methods. The flotation results showed that upon 5 × 10^−5^ mol/L Fe(CN)_6_^3−^ addition, pyrite recovery drastically decreased from about 51.3% to 8.6%, while the subsequent addition of 9.5 × 10^−4^ mol/L lead ions significantly activated pyrite with the recovery increasing from 8.6% to 91%, which demonstrated that Fe(CN)_6_^3−^ strongly depressed pyrite flotation, while lead ions completely activated pyrite in the presence of Fe(CN)_6_^3−^. Zeta potential measurement, surface analysis using Cryogenic X-ray photoelectron spectroscopy (Cryo-XPS) and electrochemical impedance spectroscopy (EIS) revealed that Fe(CN)_6_^3−^ depression was attributed to the chemical adsorption of Fe(CN)_6_^3−^ on iron sites of pyrite as Prussian Blue (Fe[Fe(CN)_6_]); however, this hydrophilic layer could be covered totally by lead ions which adsorbed on as lead hydroxide/oxide through electrostatic interactions, which resulted in the significant activation effect of lead ions. The results from this study will lead to improved flotation of gold associated with pyrite in gold flotation plants.

## 1. Introduction

In gold processing plants, the recovery of gold from pyritic gold ores involves two important steps: flotation, which separates gold and associated pyrite from other gangue minerals [1], and cyanidation, which dissolves gold in cyanide solution [2]. The tailing streams after gold cyanidation are subjected to cyanide detoxification to destroy cyanide species with the clarified water recycled back as process water. Despite cyanide detoxification, cyanide species remain in process water. Table 1 shows the commonly found residual cyanide species in process water in gold processing plants together with their stability constants [3]. These species are classified into free, weak acid dissociable (WAD) and strong acid dissociable (SAD) cyanide. SAD cyanide is more stable than free and WAD cyanide and therefore occurs more widely in process water. Among the two iron cyanide species, Fe(CN)_6_^4−^ is less stable than Fe(CN)_6_^3−^ and can be readily oxidized to form Fe(CN)_6_^3−^ in pulp [3]. 

Free cyanide has been found deleterious to pyrite flotation. Wang and Forssberg observed iron cyanide species formed after free cyanide interacting with pyrite [4]. Adams also found that free cyanide is able to reduce the surface hydrophobicity of pyrite by reacting with hydrophobic elemental sulfur or polysulfide to form hydrophilic thiocyanate [3] as shown in Reaction (1). A number of studies also showed that the presence of free cyanide inhibits xanthate collectors from adsorbing on pyrite through reducing pulp potential to hinder xanthate oxidation and adsorption [5,6,7] or replacing the adsorbed xanthate [8]. The replacing effect is explained by the stronger affinity of free cyanide than xanthate with pyrite [6].

(1) $$S^{2-}/S_{2}^{2-}/\;S_{n}^{2-}+CN^{-}\to SCN^{-}\;$$

Compared to free cyanide, the effect of iron cyanide as a main cyanide species in process water on pyrite flotation and the underpinning mechanisms are unclear. Although some researchers observed that iron cyanide depressed pyrite flotation at a small concentration in the industry [9], such an effect has not been confirmed in well-designed experiments, let alone been counteracted by any means strategically. So far, it has been uncertain whether iron cyanide depresses pyrite during flotation and, if it does, how it adsorbs on pyrite and interacts with xanthate collectors used to float pyrite and associated gold.

In fact, copper ions have been added during pyrite flotation to reduce the deleterious impact of free cyanide [10]. It is well known that copper (II) ions can adsorb on fresh pyrite, be reduced to copper (Ι) ions electrochemically and form a new copper (Ι) sulfide layer, which is called copper activation [11,12]. Guo and Peng added copper (II) ions to a complex with free cyanide, forming copper cyanide during pyrite flotation [13]. They found that copper cyanide interacts with pyrite, forming Cu(Ι)S under an oxidizing condition but Cu(Ι)_2_S under a reducing condition. This means that copper cyanide can also activate pyrite during flotation, depending on the electrochemical environment. However, copper ions have an adverse effect on gold leaching. As found by Zheng et al., the presence of copper ions during cyanidation not only slows down the gold leaching kinetic, but also demands a high cyanide consumption because copper ions strongly complex with free cyanide [14]. 

To identify an alternative to copper ions in activating pyritic gold ore flotation, Yang et al. compared copper and lead ions in improving pyrite flotation [15]. They found that lead ions highly adsorb on slightly oxidized pyrite, fully restoring pyrite floatability, while lead ions slightly adsorb on severely oxidized pyrite, activating pyrite to some extent in comparison with little activation by copper ions. Following this study, the same authors evaluated pyrite flotation with free cyanide and lead ions coexisting [16]. They reported that lead ions are able to stop free cyanide from depressing pyrite. Although free cyanide interacts with pyrite to form Fe-CN and SCN^−^ species which depress pyrite during flotation, Pb-O/OH can coat Fe-CN and SCN^−^ species and then activate pyrite. They also found that lead ions do not compete with free cyanide for adsorption sites on pyrite; instead, they adsorb on pyrite through covering the top of adsorbed free cyanide. 

Free cyanide and iron cyanide are quite different in terms of their chemical structures. It is expected that their interactions with pyrite are different no matter whether lead ions are present. Hence, this study aimed to resolve unanswered questions about pyritic gold ore flotation when iron cyanide is present. Firstly, well-defined flotation tests which simulated the industry conditions were carried out without and with the addition of iron cyanide and lead ions to identify whether iron cyanide could depress pyrite during flotation and whether lead ions could stop iron cyanide from depressing pyrite. Then the interactions between iron cyanide, lead ions and pyrite were investigated by combining Cryo-XPS, EIS analyses and Zeta potential tests under the context of pyrite flotation. While Cryo-XPS detected the surface species underpinning pyrite flotation under different conditions, EIS probed into the electrical structure of pyrite after adsorption of iron cyanide and lead ions and Zeta potential tests evidenced the formation of a type of new iron cyanide and lead species. The results from this study will recommend how to improve pyritic gold ore flotation when iron cyanide is present in process water. 

## 2. Experimental Setup

**Figure 1:**
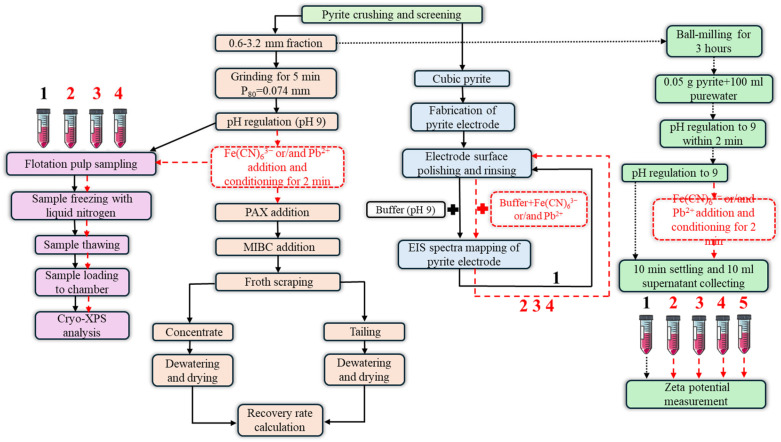
A schematic illustration of the experimental procedures in this research.

A schematic diagram of the experimental procedures was described in this research as shown in Figure 1.

### 2.1. Materials

The pyrite sample purchased from GEO Discoveries, West Gosford, Australia was crushed and the size fraction of +0.6–3.2 mm from the crushed sample was collected and preserved under −18 °C for grinding and flotation tests. This size fraction was analyzed by Inductively Coupled Plasma Mass Spectroscopy (ICP-MS) (Agilent 7900, Agilent Technologies, Santa Clara, CA, USA) and the result shown in Table 2 indicated that Fe and S were the main elements, with 45.4 and 50.5 wt.%, respectively, together with some minor elements such as Al_2_O_3_, SiO_2_ and Ti. The ICP-MS analysis result manifested a 96% purity of the selected pyrite size fraction. 

Potassium Amyl Xanthate (PAX) and Methyl Iso-Butyl Carbinol (MIBC), which are widely utilized in pyritic gold ore flotation in the industry as a collector and a frother, respectively, were used in this study. A total of 5 wt.% NaOH and 5 wt.% H_2_SO_4_ were prepared to adjust the pH. K_3_Fe(CN)_6_ and Pb(NO_3_)_2_ were selected to introduce iron cyanide and lead ions, respectively. Selecting Fe(CN)_6_^3−^ against Fe(CN)_6_^4−^ as iron cyanide was premised on the fact that Fe(CN)_6_^4−^ is more sensitive to an oxidizing condition, such as the regular flotation environment, and can be easily oxidized to Fe(CN)_6_^3−^. A pH 9.0 buffer solution used in EIS analysis was made by mixing 100 mL of 0.025 mol/L borax (Na_2_B_4_O_7_·10H_2_O) solution with 9.2 mL of 0.1 mol/L HCl solution. All of the chemicals indicated here are of analytical grade. Solutions of these chemicals were prepared daily by dissolving these chemicals in de-ionized water. All the reagents were purchased from Orica Australia Pty Ltd, Gladstone, QLD, Australia.

### 2.2. Methods

#### 2.2.1. Grinding and Flotation

A total of 100 g crushed pyrite in the size fraction of +0.6–3.2 mm and 100 mL tap water was placed in a stainless steel rod mill and ground with stainless steel rods to produce a mill discharge with 80% particles passing 106 μm. Then, the slurry in the mill was relocated into a 1.5 L flotation cell and agitated with flotation pH being adjusted to and maintained at 9.0. Where applicable, K_3_Fe(CN)_6_ and Pb(NO_3_)_2_ were added sequentially to flotation pulp at 5 × 10^−5^ and 9.5 × 10^−5^ mol/L, respectively, and each was conditioned for 2 min. The concentration of iron cyanide chosen here was the same as the concentration of free cyanide used to depress pyrite in the previous study and the same concentration of Pb(NO_3_)_2_ was also used in the previous study to activate pyrite during flotation when free cyanide was present [16]. After PAX and MIBC with a concentration of 3.3 × 10^−5^ and 5.8 × 10^−5^ mol/L were added sequentially in flotation pulp, followed by 2 and 1 min of conditioning, respectively, froth was generated by introducing air at 6 L/min and collected every 10 s. In total, four concentrates were obtained with continuous froth collection for 1.0, 2.0, 3.0 and 4.0 min. Every flotation test was repeated three times, showing an experimental error less than 4%. 

#### 2.2.2. Cryo-XPS Analysis

Cryo-XPS analysis was carried out to determine the surface products on pyrite using a KRATOS Axis Ultra XPS system (KRATOS, Manchester, UK) with a monochromatic Al Ka X-ray source, which was operated at 150 W to provide photons of 1486.6 eV. The samples for Cryo-XPS analysis were taken by a 10 mL syringe during the flotation when the slurry was mixed. These samples were frozen in liquid nitrogen as soon as they were taken. How the frozen samples were treated and loaded to the XPS system was detailed elsewhere [17]. The collecting of data and the calibration of XPS spectra as well as peak fitting using CasaXPS software (version 2.3.15) were reported in previous studies [16,18]. The XPS spectra were charge-corrected with reference to adventitious carbon contamination at 284.8 eV [19,20]. It is known that the cryogenic technique allows the direct analysis of intact fast-frozen hydrated samples, which avoids surface changes resulting from water loss during the pre-treatment of samples.

#### 2.2.3. Zeta Potential Analysis

Zeta potential tests were performed using a Malvern Zetasizer Nano ZS90 (Malvern Panalytical, Malvern, UK) to investigate the influence of Fe(CN)_6_^3−^ or/and Pb^2+^ on zeta potential of pyrite at pH 9. A total of 100 g of the prepared pyrite sample was ground using a 1 L ball mill for 3 h and the discharge sample was collected and stored in a freezer for subsequent mixing with de-ionized water. For each zeta potential test, 0.05 g sample was mixed with 100 mL de-ionized water using a magnetic stirring machine conditioning for 2 min while pH was being regulated to 9, followed by the addition of Fe(CN)_6_^3−^ or/and Pb^2+^ and another conditioning of 2 min for each reagent where applicable. Then the mixture was allowed to settle for 5 min, producing a supernatant layer from which 10mL liquid was collected for zeta potential tests. The final zeta potential value was taken as an average of three measurements. 

#### 2.2.4. EIS Analysis

EIS analysis was performed using a CHI 920D Scanning Electrochemical Microscope (CH Instruments, Bee Cave, TX, USA) to investigate the surface characteristics of pyrite after the interaction of iron cyanide with lead ions. A pyrite electrode with a surface area of about 1.56 cm^2^ was made from a pyrite specimen, hand-picked from the purchased pyrite sample. This pyrite electrode, a platinum electrode and a Ag/AgCl electrode with a solution of 3 mol/L KCl, were employed as the working, counter and reference electrodes, respectively. For EIS analysis, these three electrodes were connected in a three-electrode electrochemical cell containing 200 mL buffer solution through three specially designed holes on the electrochemical cell.

Before each EIS measurement, the surface of pyrite electrode was wet polished using 1200 grit silicon carbide, followed by rinsing with de-ionized water as reported in the literature [21]. Where iron cyanide and lead ions were added, the freshly polished pyrite electrode surface was first conditioned for 15 min in a beaker with 200 mL pH 9.0 buffer solution containing 5 × 10^−5^ mol/L K_3_Fe(CN)_6_ and/or 9.5 × 10^−5^ mol/L Pb(NO_3_)_2_. After conditioning, the pyrite electrode was taken out and rinsed gently with de-ionized water to remove the contaminated species. It was then inserted into the electrochemical cell for EIS measurement. Each EIS measurement was repeated three times with an experimental error of less than 2%. The EIS results were fitted using Zview software (version 2.0) to extract electrical parameters to understand the surface characteristics of pyrite treated under different conditions. 

## 3. Results and Discussion

### 3.1. Flotation

#### 3.1.1. Effect of Iron Cyanide on Pyrite Flotation 

**Figure 2:**
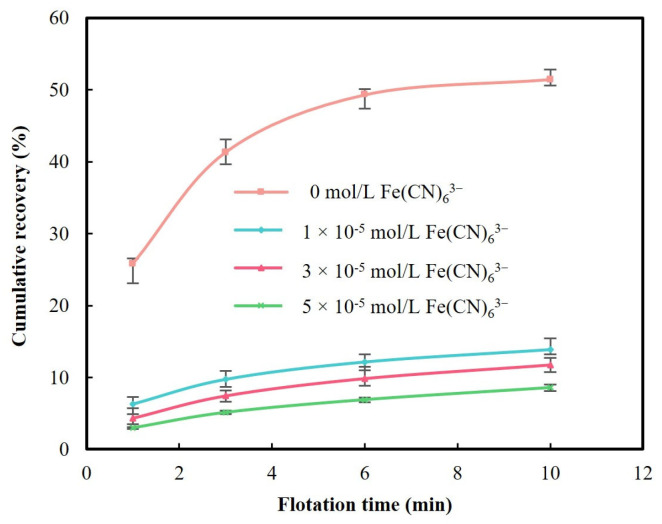
Cumulative recoveries of pyrite without and with different concentrations of Fe(CN)_6_^3−^ at pH 9.0.

To understand whether Fe(CN)_6_^3−^ depressed pyrite during flotation, pyrite flotation tests were carried out at pH 9.0 without and with Fe(CN)_6_^3−^ addition. Figure 2 shows the change of cumulative pyrite recovery within 10 min as the Fe(CN)_6_^3−^ concentration increases. 

In Figure 2, pyrite recovery was low at pH 9.0 even without Fe(CN)_6_^3−^, reaching about 51.5% at the completion of flotation. It has been known that the oxidation of pyrite is facilitated under alkaline conditions, forming hydrophilic ferric hydroxide on the surface [22]. This is unfavorable for pyrite flotation in general. However, the addition of a high concentration of PAX can still promote pyrite flotation due to electrochemical reduction of ferric hydroxide to much more soluble ferrous hydroxide, coupled with oxidation and adsorption of PAX [23]. 

Figure 2 also suggests that the presence of Fe(CN)_6_^3−^ exacerbated pyrite flotation. As can be noticed, pyrite recovery decreased to 13.0% after the addition of 1 × 10^−5^ mol/L Fe(CN)_6_^3−^. The addition of 5 × 10^−5^ mol/L Fe(CN)_6_^3−^ further decreased pyrite recovery to only 8.6%. Obviously, Fe(CN)_6_^3−^ depressed pyrite significantly at pH 9.0. Yang et al. reported that pyrite recovery decreased from 51.5% without the addition of any cyanide species to 33.1% with the addition of 5 × 10^−5^ mol/L CN^−^ [16]. Obviously, iron cyanide depressed pyrite more than free cyanide. 

The mechanisms responsible for free cyanide in depressing pyrite have been reported and the formation of Fe-CN species on pyrite upon free cyanide interacting with pyrite has been attributed to the reduced adsorption of collector xanthate and subsequent pyrite depression [7,24]. In the previous study, it was found that CN^−^ can also interact with reactive sulfur species on pyrite, forming SCN^−^ species; therefore, the hydrophobic sulfur species are removed from the surface, contributing to decreased surface hydrophobicity [16]. Unlike free cyanide, the interaction of pyrite with Fe(CN)_6_^3−^ has not been studied. In the present study, Cryo-XPS analysis was conducted to identify the surface products on pyrite upon Fe(CN)_6_^3−^ interacting with pyrite to investigate the mechanisms responsible for the strong depressing effect of Fe(CN)_6_^3−^ on pyrite. The results are presented in Section 3.2. 

#### 3.1.2. Effect of Lead Ions on Pyrite Flotation in the Presence of Iron Cyanide 

**Figure 3:**
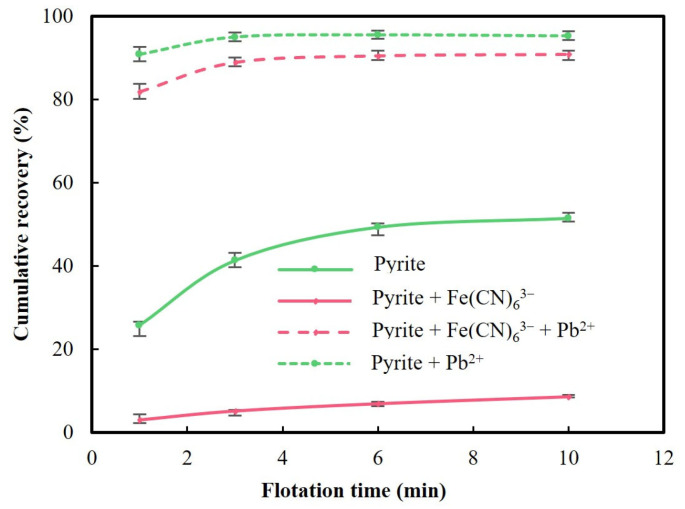
Cumulative recoveries of pyrite from the flotation at pH 9.0 without Fe(CN)_6_^3−^ and Pb^2+^, with only 5 × 10^−5^ mol/L Fe(CN)_6_^3−^, with only 9.5 × 10^−4^ mol/L Pb^2+^ and with both 5 × 10^−5^ mol/L Fe(CN)_6_^3−^ and 9.5 × 10^−4^ mol/L Pb^2+^.

After identifying the depression role of Fe(CN)_6_^3−^ during pyrite flotation, pyrite flotation tests were carried out with both Fe(CN)_6_^3−^ and Pb^2+^ to investigate whether the strong depressing effect of Fe(CN)_6_^3−^ in pyrite flotation could be counteracted by lead ions. Figure 3 shows the cumulative recovery of pyrite with both Fe(CN)_6_^3−^ and Pb^2+^ in 10 min. The cumulative recovery curves of pyrite without Fe(CN)_6_^3−^ and Pb^2+^, with Fe(CN)_6_^3−^ and with Pb^2+^ were also plotted in Figure 3 for comparison. 

As shown in Figure 3, pyrite recovery was 95.2% when 9.5 × 10^−4^ mol/L Pb^2+^ was present in comparison with a pyrite recovery of 51.5% when Pb^2+^ was not present. Obviously, lead ions significantly activated pyrite. Such activation in alkaline conditions has been attributed to lead oxide/hydroxide adsorbed on pyrite driven by electrostatic interactions, which subsequently enhances the adsorption of xanthate [15,19,25,26,27,28]. Xanthate adsorbs on lead-activated pyrite in the form of lead xanthate, proceeding finally to dixanthogen, and dixanthogen is responsible for a strong activation effect [15,19,25,26,27,28]. 

Figure 3 also shows a pyrite recovery of 91.0% from the flotation with the addition of 5 × 10^−5^ mol/L Fe(CN)_6_^3−^ and 9.5 × 10^−4^ mol/L Pb^2+^ in comparison with a pyrite recovery of 8.6% from the flotation with the addition of only 5× 10^−5^ mol/L Fe(CN)_6_^3−^. It is clear that 9.5 × 10^−4^ mol/L Pb^2+^ not only fully offset the depression effect of 5 × 10^−5^ mol/L Fe(CN)_6_^3−^ on pyrite, but also almost completely activated the pyrite. In the previous study, 76.8% pyrite recovery was reported from the flotation with the addition of 5 × 10^−5^ mol/L CN^−^ and 9.5 × 10^−4^ mol/L Pb^2+^ [16]. This indicates that the same amount of lead ions, 9.5 × 10^−4^ mol/L, was more efficient in activating pyrite when coexisting with 5 × 10^−5^ mol/L Fe(CN)_6_^3−^ than 5 × 10^−5^ mol/L CN^−^, although Fe(CN)_6_^3−^ depressed pyrite more than CN^−^.

Pyrite activation by lead ions during flotation with free cyanide coexisting has been attributed to lead oxide/hydroxide adsorbed on Fe-CN and SCN^−^ species formed on pyrite, but lead oxide/hydroxide preferentially adsorbs on SCN^−^ species, which explains the limited capability of lead ions in activating pyrite depressed by free cyanide [16]. Since lead ions showed different effects on pyrite after pyrite was depressed by CN^−^ and Fe(CN)_6_^3−^, it was expected that lead ions interacted differently with the two types of cyanide species on pyrite. The interaction of Fe(CN)_6_^3−^ with pyrite was studied below.

### 3.2. Surface Species on Pyrite

Cryo-XPS and zeta potential analysis were conducted to determine the surface products on pyrite which underpinned pyrite flotation with only Fe(CN)_6_^3−^ and both Fe(CN)_6_^3−^ and lead ions coexisting. 

#### 3.2.1. Surface Species Underpinning Pyrite Depression by Fe(CN)_6_^3−^

Pyrite depression by Fe(CN)_6_^3−^ during flotation should stem from the interaction of Fe(CN)_6_^3−^ with pyrite. 

**Figure 4:**
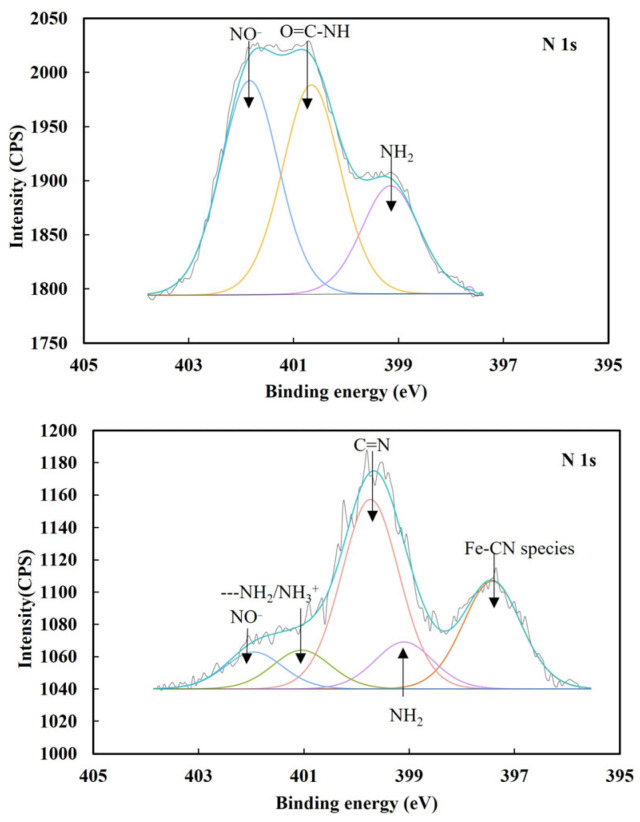
XPS N 1s spectra obtained from pyrite without (**up**) and with (**down**) 5 × 10^−5^ mol/L Fe(CN)_6_^3−^.

Figure 4 presents the XPS N 1s spectra obtained from pyrite without and with Fe(CN)_6_^3−^, from which the cyanide species formed could be inferred.

As presented in Figure 4, without Fe(CN)_6_^3−^, the N 1s spectrum of pyrite displayed three peaks at 399.1, 400.7 and 401.9 eV, which were ascribed to NH_2_, O=C-NH and NO^−^, respectively [29]. These N species originated from surface contamination. However, with 5 × 10^−5^ mol/L Fe(CN)_6_^3−^, the N 1s spectrum showed five peaks at 397.4, 399.1, 399.7, 401.0 and 402.0 eV. NO^−^ is an oxidized N species and its N-O bond is normally located at around 402.2 eV [30]. Therefore, the peak at 402.0 eV was ascribed to N bonded in NO^−^ species. The characteristic N peak for nitrogen bonded to carbon in cyanide groups is usually located at 397.2–397.9 eV [31,32]. Therefore, the new peak at 397.3 eV was ascribed to N bonded in Fe-CN species. 

Table 3 presents the atomic concentrations of nitrogen bonded in nitrogen species on pyrite. It can be found that the interaction of 5 × 10^−5^ mol/L Fe(CN)_6_^3−^ with pyrite formed 1.57 at.% N associated with Fe-CN species which underpinned pyrite depression by 5 × 10^−5^ mol/L Fe(CN)_6_^3−^ in this study. It has been reported that Fe(CN)_6_^3−^ can interact with ferrous ions to generate Prussian Blue (Fe_4_[Fe(CN)_6_]_3_), while Fe(CN)_6_^4−^ can interacted with ferric ions to generate Turnbull’s Blue (Fe_3_[Fe(CN)_6_]_2_) in a Fe–S–CN–H_2_O system [7,25], which was supported by thermodynamic calculations [4]. In fact, they have the same chemical formula, Fe[Fe(CN)_6_]. Therefore, in this study, it was reasonable to expect that the hydrophilic Fe-CN compound, Fe[Fe(CN)_6_], formed on pyrite upon the chemical interaction of Fe(CN)_6_^3−^ with iron sites then prevented PAX from being oxidized and adsorbing on pyrite, resulting in the depression of pyrite. 

#### 3.2.2. Surface Species Underpinning Pyrite Activation by Lead Ions 

**Figure 5:**
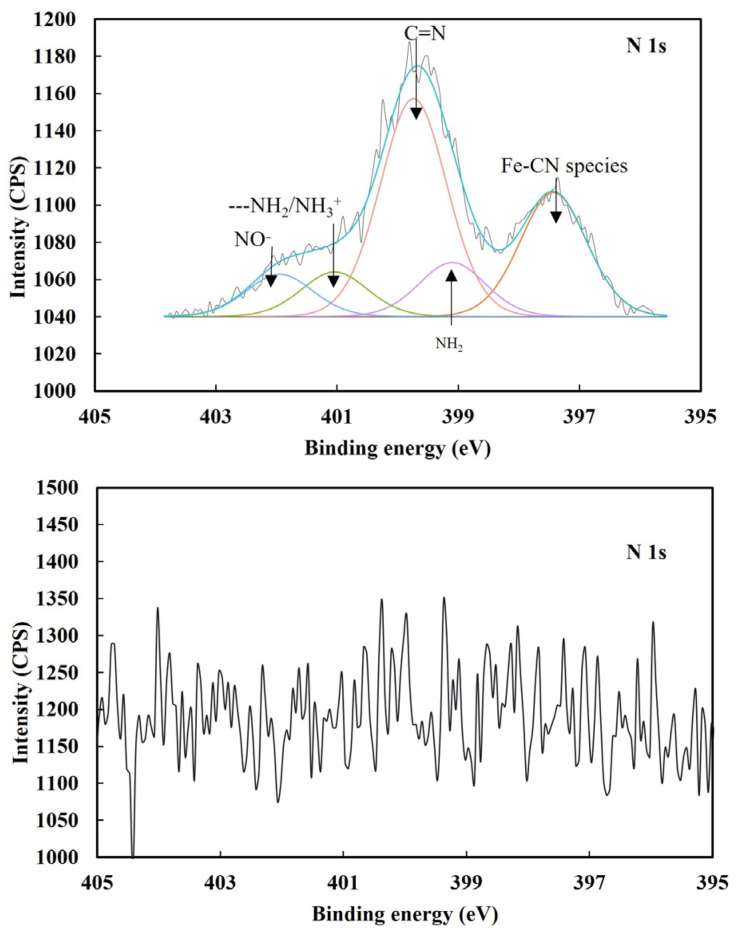
XPS N 1s spectra of pyrite after treatment with only 5 × 10^−5^ mol/L Fe(CN)_6_^3−^ and both 5 × 10^−5^ mol/L Fe(CN)_6_^3−^ and 9.5 × 10^−4^ mol/L Pb^2+^.

To identify the surface species which were responsible for lead activation for iron cyanide depressed pyrite, and how lead ions interact with iron cyanide on pyrite surface, the XPS N 1s spectra of pyrite treated by 5 × 10^−5^ mol/L Fe(CN)_6_^3−^ and by both 5 × 10^−5^ mol/L Fe(CN)_6_^3−^ and 9.5 × 10^−4^ mol/L Pb^2+^ were obtained and presented in Figure 5. 

From Figure 5, it can be found that Fe-CN species formed on pyrite treated by 5 × 10^−5^ mol/L Fe(CN)_6_^3−^, but the Fe-CN species disappeared after pyrite was treated by both 5 × 10^−5^ mol/L Fe(CN)_6_^3−^ and 9.5 × 10^−4^ mol/L Pb^2+^. Although it was not clear why the Fe-CN species disappeared when Pb^2+^ was present with Fe(CN)_6_^3−^ at this stage of analysis, this phenomenon did explain the high pyrite recovery when both lead ions and Fe(CN)_6_^3−^ were present at the same time. 

**Figure 6:**
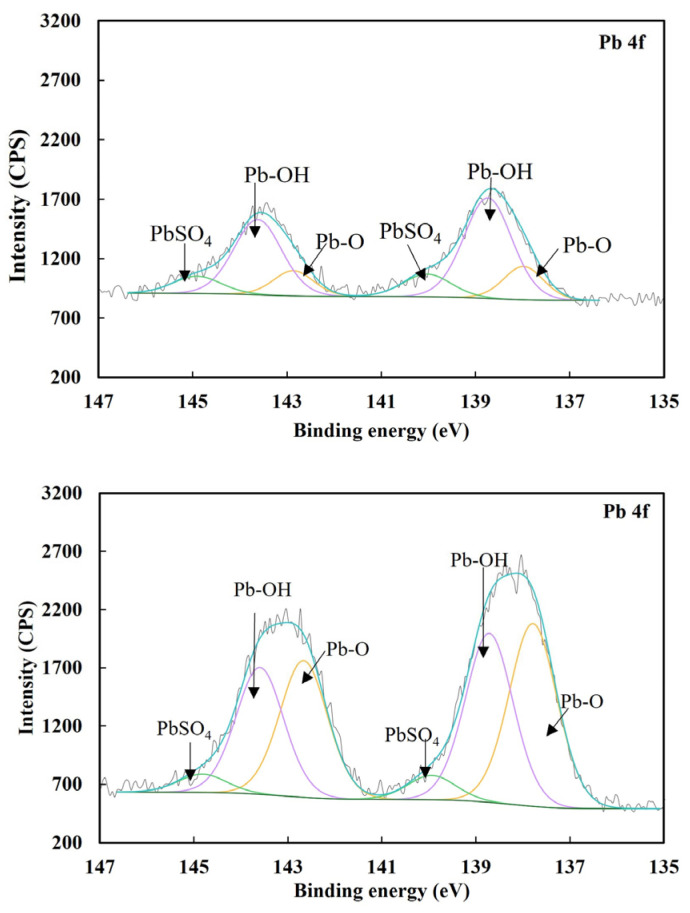
Pb 4f spectra of pyrite with only 9.5 × 10^−4^ mol/L Pb^2+^ and both 5 × 10^−5^ mol/L Fe(CN)_6_^3−^ and 9.5 × 10^−4^ mol/L Pb^2+^.

Figure 6 shows the Pb 4f spectra of pyrite treated by only 9.5 × 10^−4^ mol/L Pb^2+^ and both 5 × 10^−5^ mol/L Fe(CN)_6_^3−^ and 9.5 × 10^−4^ mol/L Pb^2+^, and Table 4 shows the atomic concentrations of lead bonded in lead species under these two conditions. Figure 6 indicates that in the presence of only Pb^2+^, three lead peaks occurred at 137.9, 138.8 and 140.2 eV. The peak at 137.9 eV was ascribed to the lead bonded in Pb-O [33] or Pb-S [34], but Pb-O was favored at an alkaline pH [35]. The peaks at 138.8 and 140.2 eV were ascribed to the lead bonded in Pb-OH [36] and PbSO_4_ [37], respectively. Table 4 indicates 1.58 at.% lead on pyrite, which was responsible for pyrite activation by 9.5 × 10^−4^ mol/L Pb^2+^. Pecina et al. proposed that Pb-O/OH is the major lead species that activates pyrite by catalyzing the adsorption of xanthate [38].

When pyrite was treated by both 5 × 10^−5^ mol/L Fe(CN)_6_^3−^ and 9.5 × 10^−4^ mol/L Pb^2+^, three Pb 4f peaks appeared at 137.8, 138.7 and 139.9 eV, ascribed to the lead bounded in Pb-O, Pb-OH and PbSO_4_, respectively. In fact, the types of lead species adsorbed on pyrite after the treatment with only Pb^2+^ and both Fe(CN)_6_^3−^ and Pb^2+^ were the same. However, it was interesting to find that the total lead concentration on pyrite after the treatment with both 5 × 10^−5^ mol/L Fe(CN)_6_^3−^ and 9.5 × 10^−4^ mol/L Pb^2+^ increased to 3.37 at.% from 1.85 at.% on pyrite. In the previous study, CN^−^ shifts the zeta potential of pyrite negatively upon interacting with pyrite, thus enhancing the adsorption of lead species with positive charges [16]. In that study, the lead concentration on pyrite increased from 1.85 at.% after the surface was treated by 9.5 × 10^−4^ mol/L Pb^2+^ to only 2.16 at.% after the surface was treated by both 5 × 10^−5^ mol/L CN^−^ and 9.5 × 10^−4^ mol/L Pb^2+^. Obviously, the same type of interaction occurred here when CN^−^ was replaced by Fe(CN)_6_^3−^. However, Fe(CN)_6_^3−^ could adsorb on pyrite and make pyrite more negative than CN^−^, promoting more lead species to adsorb on the top through electrostatic attraction. It was unlikely that lead ions replaced Fe(CN)_6_^3−^ on pyrite, given a greater amount of lead species adsorbed when lead ions and Fe(CN)_6_^3−^ coexisted, and the stronger interaction of pyrite with Fe(CN)_6_^3−^ than with lead ions. While the interaction between Fe(CN)_6_^3−^ and lead ions was studied further in the next section, the coating of Fe(CN)_6_^3−^ by lead ions on pyrite was responsible for lead ions counteracting Fe(CN)_6_^3−^ depression on pyrite flotation. 

It should be noted that free cyanide interacting with pyrite forms two cyanide species, Fe-CN and SCN^−^, and the formation of SCN^−^ species limits pyrite activation by lead ions [16]. Here, only Fe-CN species formed after the interaction of Fe(CN)_6_^3−^ with pyrite, and therefore, lead ions could potentially fully activate pyrite. This is consistent with the same level of pyrite recoveries achieved from the flotation with the addition of only 9.5 × 10^−4^ mol/L Pb^2+^ and both 5 × 10^−5^ mol/L Fe(CN)_6_^3−^ and 9.5 × 10^−4^ mol/L Pb^2+^ as shown in Figure 3.

#### 3.2.3. Zeta Potential of Pyrite Surfaces upon Addition of Lead Ions and/or Fe(CN)_6_^3−^

The zeta potential of pyrite with lead ions, Fe(CN)_6_^3−^, lead ions and Fe(CN)_6_^3−^, and Fe(CN)_6_^3−^ and lead ions were tested and the result was presented in Table 5. The zeta potential value of pyrite at pH 9 was also listed for comparison. 

Table 5 showed that without any reagent addition, the pyrite surface was negatively charged with a value of −10.37 mV at pH 9, which reflected the surface oxidation and the formation of ferric hydroxide at alkaline pH values. Upon Fe(CN)_6_^3−^ addition, the potential value was dramatically shifted to a more negative −37.36mV, demonstrating the chemical adsorption of Fe(CN)_6_^3−^ on iron sites of pyrite and the resulted formation of Prussian Blue, the zeta potential of which has been reported around −37.5~−33 mV [39,40,41,42]. Upon the addition of lead ions, the potential value significantly increased to 31.02 mV; this means that lead species chemically adsorbed on the pyrite surface. It is also interesting to note that changing the addition order of lead ions and Fe(CN)_6_^3−^ has little influence on zeta potential, and this suggests that subsequent adsorption of lead ions cannot replace the already formed Fe-CN species and vice versa, which means lead species and Fe(CN)_6_^3−^ will not compete with each other on the pyrite surface when coexisting in pyrite flotation. 

### 3.3. EIS of Pyrite Surfaces 

EIS analysis was performed to identify the impedance of pyrite treated by only Fe(CN)_6_^3−^, both Fe(CN)_6_^3−^ and Pb^2+^, and only Pb^2+^ with an objective of identifying the interaction between Fe(CN)_6_^3−^ and Pb^2+^ on pyrite. 

**Figure 7:**
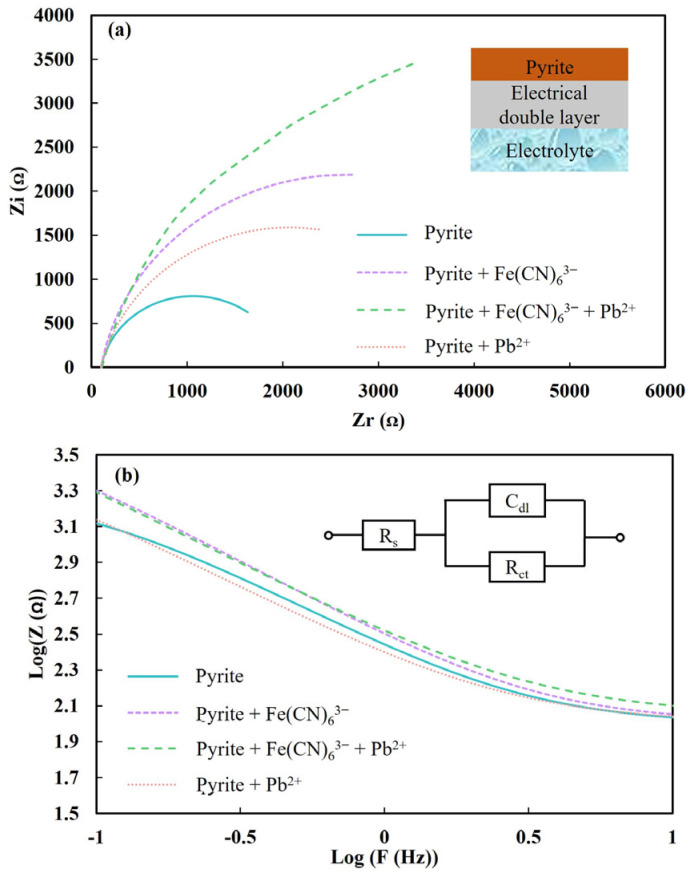
EIS spectra of pyrite after treatment without and with 5 × 10^−5^ Fe(CN)_6_^3−^, with both 5 × 10^−5^ Fe(CN)_6_^3−^ and 9.5 × 10^−4^ mol/L Pb^2+^ and with only 9.5 × 10^−4^ mol/L Pb^2+^ in the form of Nyquist (**a**) and Bode plots (**b**).

Figure 7 shows the EIS results. The measured EIS spectra were modeled using Z-view software (version 2.0) and the modelling parameters are shown in Table 6. 

Figure 7a shows this electrical double layer of pyrite in an electrolyte solution consisting of two planes with equal and opposite charges like a capacitor. The pyrite electrode/electrolyte interface can be represented by a simple electrical circuit, as shown in Figure 7b. The electrical circuit is made of a solution resistance (R_s_) and a parallel circuit comprising the double layer capacitance (C_dl_) and the charge transfer resistance (R_ct_). Considering surface inhomogeneity and roughness, the double layer capacitor of pyrite was simulated by a constant phase element (CPE). R_ct_, R_s_ and C_dl_ values were determined from the extracted modeling parameters. The n value shown in Table 6 is another extracted parameter indicating the roughness of the electrode surface. Normally, n varies between 0.5 to 1. The higher the n value, the more homogeneous the electrode surface, and the CPE tends to be an ideal capacitor when n approaches 1 [43]. The modeled spectra are presented in the form of Nyquist plot showing the imaginary impedance (Z_i_) against the real impedance (Z_r_) (Figure 7a), and also in the form of Bold plots showing the logarithm of the absolute magnitude of impedance (Z) against the logarithm of frequency (F) (Figure 7b). 

The Nyquist plots in Figure 7a show incomplete semicircles with different radii. For a perfect capacitor with the n value being 1, the radius is proportional to R_ct_. Figure 7a shows a greater radius with iron cyanide than without Fe(CN)_6_^3−^. This indicates the charge transfer resistance of pyrite increasing upon the chemical interaction of Fe(CN)_6_^3−^ with pyrite. This can also be seen from the R_ct_ values in Table 6. It has been reported that electron transfer is mainly controlled by charge transfer resistance [21]. The higher R_ct_ shows that it would be more difficult for electrons to penetrate through the surface layer, and also that the pyrite electrode thickness increased upon Fe(CN)_6_^3−^ adsorption based on the premise that a higher R_ct_ relates to a higher electrode thickness [43]. 

In Bode plots, impedance is inversely proportional to capacitance in the intermediate frequency domain. The intermediate region of the Bode plots in Figure 7b show higher impedance with Fe(CN)_6_^3−^ than without it. This is reflected by the lower capacitance value with Fe(CN)_6_^3−^ as shown in Table 6. The n values for pyrite without and with Fe(CN)_6_^3−^ were pretty much the same, being 0.89 and 0.90, respectively. The n value for pyrite without Fe(CN)_6_^3−^ was determined by the coverage of iron hydroxide, the main pyrite oxidation product [21], while the similar n value for pyrite with Fe(CN)_6_^3−^ was governed by Fe-CN species formed as discussed previously, probably Fe[Fe(CN)_6_], called Prussian Blue, due to the zeta potential of the Fe(CN)_6_^3−^–adsorbed pyrite surface being similar to that of pure Prussian Blue nanoparticles. 

The R_ct_ of pyrite with lead ions was greater than that without lead ions, in line with the different radii of incomplete semicircles in the Nyquist plots. This change was caused by lead ions adsorbed on pyrite with the n value decreasing slightly to 0.86. Interestingly, the R_ct_ of pyrite with both lead ions and Fe(CN)_6_^3−^ increased significantly to 9438 ꭥ from 3952 ꭥ with only lead ions, or from 5149 ꭥ with only Fe(CN)_6_^3−^. As mentioned previously, a higher R_ct_ value relates to a higher electrode thickness [43]. Therefore, it could be concluded that lead species co-adsorbed with Fe(CN)_6_^3−^ species on pyrite when lead ions and Fe(CN)_6_^3−^ coexisted, which was supported by the similar zeta potential values (−3.04mV for adding Fe(CN)_6_^3−^ first, then lead ions, and −4.67mV for adding them in the reverse order when changing the addition order of lead ions and Fe(CN)_6_^3−^ as shown in Table 5. Furthermore, the n values for pyrite without both lead ions and Fe(CN)_6_^3−^ decreased upon the addition of lead ions, from 0.89 to 0.85; similarly, the n value of pyrite with Fe(CN)_6_^3−^ also decreased upon the addition of lead ions, from 0.90 to 0.86. This supports the coating of lead ions on pyrite surface regardless of Fe(CN)_6_^3−^ presence. 

Overall, this study discovered that the interaction of Fe(CN)_6_^3−^ with pyrite probably yields hydrophilic Prussian Blue and that lead ions can co-adsorb with Fe(CN)_6_^3−^ as a lead hydroxide/oxide covering on top of the Prussian Blue regardless of the addition order of Fe(CN)_6_^3−^.

**Figure 8:**
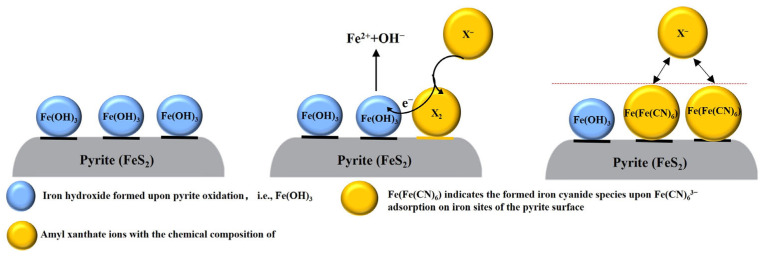
A schematic of the pyrite surface and its interaction with PAX (**left and middle**) (adapted from a journal article by Valdivieso et al. (2005) [23]) and a speculative diagram expressing the possible influence of Fe(CN)_6_^3−^ on pyrite flotation with PAX at pH 9.0 (**right**).

Figure 8 shows the schematic illustration of the flotation mechanism of pyrite covered by ferric hydroxide with xanthate collector PAX [23], and the reasonable speculation about how hydrophilic Fe[Fe(CN)_6_] formation influenced xanthate adsorption. As presented in Figure 8 (left), ferric hydroxide was generated on pyrite at pH 9.0 as a result of pyrite oxidation. It almost homogeneously covered the surface in the absence of Fe(CN)_6_^3−^, supported by the high surface homogeneity parameter n obtained from the EIS analysis. Despite the ferric hydroxide on the pyrite, PAX (X^−^) could still be oxidized to dixanthogen (X_2_), accompanied by the reduction of ferric hydroxide, leading to the generation of dixanthogen on the pyrite [23] as indicated in Figure 8 (middle). The generation of dixanthogen promoted pyrite flotation to some extent, as evidenced in Figure 2. With the addition of Fe(CN)_6_^3−^, this iron cyanide species chemically interacted with ferric ions in ferric hydroxides on pyrite to form stable Fe[Fe(CN)_6_] as indicated in Figure 8 (right), resulting the zeta potential of pyrite resembling that of Prussian Blue nano-particles reported in the literature. The formation of Fe[Fe(CN)_6_] also almost homogeneously covered the surface, rendering it hydrophilic; therefore, it is reasonable to speculate that Fe[Fe(CN)_6_] blocks PAX from interacting with pyrite, leading to depressed pyrite during flotation. 

**Figure 9:**
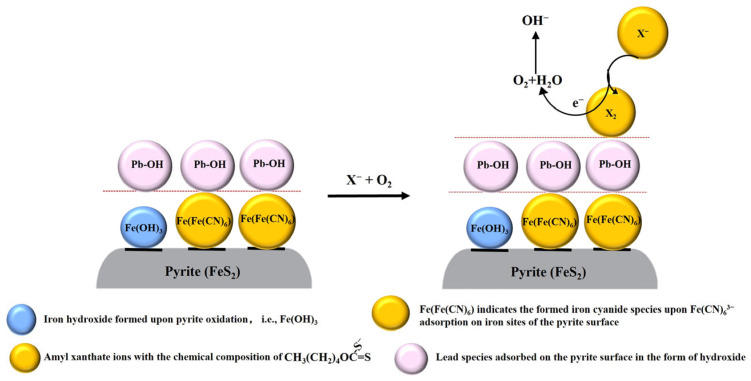
A schematic of the interaction between lead ions and Fe(CN)_6_^3−^ on pyrite and the speculative mechanism of the influence of this interaction on PAX adsorption at pH 9.0.

This study also discovered the interaction of Fe(CN)_6_^3−^ with lead ions. In this research, firstly, Fe(CN)_6_^3−^ was added, resulting in the formation of Fe[Fe(CN)_6_], which did not inhibit subsequent lead adsorption; on the contrary, it enhanced lead adsorption according to the lead atomic concentration values shown in Table 4. This enhancement was associated with electrostatic attraction rather than chemical interaction between lead ions and Fe[Fe(CN)_6_], considering the fact that if chemical interaction of lead ions with Fe[Fe(CN)_6_] occurred, the adsorbed lead species in the presence of Fe(CN)_6_^3−^ would be different from those in the absence of Fe(CN)_6_^3−^. In fact, it has been found that the type of adsorbed lead species without Fe(CN)_6_^3−^ was the same as that with Fe(CN)_6_^3−^. The similar zeta potential of pyrite when changing the addition order of lead ions and Fe(CN)_6_^3−^ also showed that lead ions do not chemically interact with Fe(CN)_6_^3−^. The decreased n value upon lead species adsorption showed that lead hydroxide/oxide can adsorb on top of it at pH 9.0, as indicated in Figure 9 (left). All of the above evidence supports that Fe(CN)_6_^3−^ will not chemically compete with lead ions for the pyrite surface; instead, Fe(CN)_6_^3−^ can enhance lead adsorption through increased electrostatical attraction, leading to coverage of hydrophilic Fe[Fe(CN)_6_] by lead hydroxide/oxide to induce activation. Besides that, it can also be speculated that lead hydroxide/oxide adsorption in the presence of Fe(CN)_6_^3−^ can enhance PAX adsorption at pH 9.0 through replacement of OH^−^ in Pb-OH by X^−^. During flotation, PAX may adsorb on the lead hydroxide/oxide-covered pyrite as lead xanthate coordinates and the xanthate end will be oxidized to dixanthogen, coupled with oxygen reduction, as shown in Figure 9 (right), rendering pyrite highly floatable. The schematic illustration of the interaction of lead ions with Fe(CN)_6_^3−^ on pyrite is shown in Figure 9 (left), together with the speculative influence of lead and iron cyanide interaction on PAX adsorption in Figure 9 (right). 

## 4. Conclusions

The flotation results showed that iron cyanide depressed pyrite during flotation greatly, while lead ions completely offset the depression role of iron cyanide and fully activate pyrite. 

The new N 1s peak identified in N 1s spectra and the dramatically increased R_ct_ value of pyrite with iron cyanide compared with that of pyrite only demonstrated that iron cyanide chemically interacts with the pyrite surface, forming a new cyanide species. It turns out that this new cyanide species was Prussian Blue, since the zeta potential value of pyrite with both iron cyanide and lead ions was in the range of that of hydrophilic Prussian Blue nano-particles. Therefore, iron cyanide can greatly depress pyrite flotation through the strong chemical interaction of iron cyanide with surface iron sites (ferric hydroxide), forming a hydrophilic Prussian Blue, rendering the pyrite surface hydrophilic and possibly making it difficult for xanthate ions to adsorb on. 

Similar zeta potential values of pyrite when changing the addition order of lead ions and iron cyanide showed that lead ions do not chemically interact or compete with iron cyanide. The Pb 4f spectra of pyrite with lead ions in the absence and presence of iron cyanide both found the adsorption of lead species as lead hydroxide/oxide. It has been well known that lead activation of pyrite proceeds through the adsorption of lead ions as a lead hydroxide/oxide layer which catalyzes xanthate adsorption. Therefore, it can be concluded that despite the fact that lead ions cannot chemically interact with iron cyanide, lead ions can electrostatically adsorb on iron-cyanide-depressed pyrite surfaces as a lead hydroxide/oxide layer, which was supported by the dramatic change of the zeta potential value towards the positive direction upon lead addition regardless of iron cyanide presence. This lead adsorption increased the surface roughness and charge transfer resistance values from EIS results, indicating that lead covers the Prussian Blue-causing surface with both lead and iron cyanide, causing it to be rougher and thicker than that covered with only iron cyanide.

The results of this study can guide the gold industry to minimize the deleterious impact of iron cyanide on pyritic gold ore flotation.

## Figures and Tables

**Table 1:** Residual cyanide species commonly found in process water in gold processing plants together with their stability constants [3].

Category	Species	LogK_n_
Free cyanide	CN^−^	-
	HCN	9.2
WAD	Cd(CN)_4_^2−^	17.9
	Zn(CN)_4_^3−^	19.6
	Cu(CN)_2_^−^	16.3
	Cu(CN)_3_^2−^	21.6
	Cu(CN)_4_^3−^	23.1
	Ni(CN)_4_^2−^	30.2
	Ag(CN)_2_^−^	20.5
SAD	Fe(CN)_6_^4−^	35.4
	Fe(CN)_6_^3−^	43.6
	Co(CN)_6_^3−^	64.0
	Hg(CN)_4_^2−^	39.0
	Au(CN)_2_^−^	38.3

**Table 2:** Compositional elements identified in pyrite fraction of +0.6–3.2 mm by ICP-MS analysis.

Sample	Elements Identified (wt.%)								
	Fe	S	Cu	Bi	Pb	Al_2_O_3_	SiO_2_	Ti	Zn
Pyrite (0.6–3.2 mm)	45.4	50.5	0.1	0.02	0.13	0.03	0.35	0.04	0.05

**Table 3:** Atomic concentrations of nitrogen bonded in nitrogen species on pyrite after treatment without and with 5 × 10^−5^ mol/L Fe(CN)_6_^3−^.

Samples	Species	N 1s B.E. (eV)	Concentration (at.%)
Pyrite	NH_2_	399.1	1.29
	O=C-NH	400.7	2.39
	NO^−^	401.9	2.51
Pyrite + Fe(CN)_6_^3−^	Fe-CN species	397.4	1.57
	NH_2_	399.1	0.68
	C=N	399.7	2.76
	---NH_2_/NH_3_^+^	401.0	0.57
	NO^−^	402.0	0.54

**Table 4:** Atomic concentrations of lead bonded in lead species on pyrite after treatment with only 9.5 × 10^−4^ mol/L Pb^2+^ and both 5 × 10^−5^ mol/L Fe(CN)_6_^3−^ and 9.5 × 10^−4^ mol/L Pb^2+^.

Sample	Species	Pb 4f_7/2_ B.E. (eV)	Concentration (at.%)
Pyrite + Pb^2+^	Pb-O	137.9	0.29
	Pb-OH	138.7	1.05
	PbSO_4_	139.8	0.24
Pyrite + Fe(CN)_6_^3−^ + Pb^2+^	Pb-O	137.8	1.64
	Pb-OH	138.7	1.52
	PbSO_4_	139.9	0.21

**Table 5:** Zeta potential values of tested samples in this study.

Sample	Pyrite	Pyrite + Fe(CN)_6_^3−^	Pyrite + Pb + Fe(CN)_6_^3−^	Pyrite + Fe(CN)_6_^3−^ + Pb	Pyrite + Pb
Zeta potential value (mV)	−10.37	−37.36	−3.40	−4.67	31.02

**Table 6:** The extracted modeling parameters of the equivalent circuits for pyrite after treatment without and with 5 × 10^−5^ mol/L Fe(CN)_6_^3−^, with both 5 × 10^−5^ mol/L Fe(CN)_6_^3−^ and 9.5 × 10^−4^ Pb^2+^ and with only 9.5 × 10^−4^ Pb^2+^.

Sample	R_s_ (ꭥ)	R_ct_ (ꭥ)	CPE(μF)	*n*
Pyrite	98.5	1928	818	0.89
Pyrite + Fe(CN)_6_^3−^	101.4	5149	676	0.90
Pyrite + Pb^2+^	99.95	3952	986	0.86
Pyrite + Fe(CN)_6_^3−^ + Pb^2+^	110.4	9438	729	0.85

## Data Availability

The data presented in this study are available on request from the corresponding author.
